# Enhanced Bonding Strength of Hydrophobically Modified Gelatin Films on Wet Blood Vessels

**DOI:** 10.3390/ijms15022142

**Published:** 2014-01-29

**Authors:** Keiko Yoshizawa, Tetsushi Taguchi

**Affiliations:** 1 Graduate School of Pure and Applied Sciences, University of Tsukuba, 1-1-1 Tennodai, Tsukuba, Ibaraki 305-8577, Japan; E-Mail: YOSHIZAWA.Keiko@nims.go.jp; 2 Biomaterials Unit, Nano-Life Field, International Center for Materials Nanoarchitectonics (MANA), National Institute for Materials Science, 1-1 Namiki, Tsukuba, Ibaraki 305-0044, Japan

**Keywords:** hydrophobic group, gelatin, film, adhesive, thermal crosslinking

## Abstract

The bonding behavior between hydrophobically modified alkaline-treated gelatin (hm-AlGltn) films and porcine blood vessels was evaluated under wet conditions. Hexanoyl (Hx: C_6_), decanoyl (Dec: C_10_), and stearyl (Ste: C_18_) chlorides were introduced into the amino groups of AlGltn to obtain HxAlGltn, DecAlGltn, and SteAlGltn, respectively, with various modification percentages. The hm-AlGltn was fabricated into films and thermally crosslinked to obtain water-insoluble films (t-hm-AlGltn). The 42% modified t-HxAlGltn (t-42HxAlGltn) possessed higher wettability than the 38% modified t-DecAlGltn (t-38DecAlGltn) and the 44% modified t-SteAlGltn (t-44SteAlGltn) films, and the t-42HxAlGltn film showed a high bonding strength with the blood vessel compared with all the hm-AlGltn films. Histological observations indicated that t-42HxAlGltn and t-38DecAlGltn remained on the blood vessel even after the bonding strength measurements. From cell culture experiments, the t-42HxAlGltn films showed significant cell adhesion compared to other films. These findings indicate that the Hx group easily interpenetrated the surface of blood vessels and effectively enhanced the bonding strength between the films and the tissue.

## 1. Introduction

Wound closure is one of the fundamental requirements in surgical operations. A suture is usually employed to close the wounded region. However, the use of sutures prolongs operation time and is not suitable for some complicated areas such as the junction site of a blood vessel and a lung. Therefore, tissue adhesives have been developed and used in the clinical field for shortening the operation time and for the closure of wounds with complicated structures [1]. However, these adhesives still possess some disadvantages in terms of bonding strength and biocompatibility [2–11]. For example, TachoSil^®^ (CSL Behring K.K., Tokyo, Japan) is a common clinically available adhesive composed of equine collagen and fibrin; it, however, has several shortcomings. Xenogeneic product collagen and allogeneic fibrin may result in infection, and the fibrin-induced adhesivity is not very strong. Especially in terms of bonding strength, adhesives bonded on soft tissues are exposed to adverse environments because about 70% of our body is made of water. In addition, body fluids such as blood plasma and lymph fluid spill out from the wound area after a surgical operation.

To overcome these obstacles, molecular design of tissue adhesive materials that show good bonding behavior even on moisture-containing tissues is required. We recently developed a hydrophobically modified gelatin (hm-Gltn)-based adhesive and showed that the resulting hm-Gltn adhesive with a low modification percentage formed a stronger bond on porcine arterial media as compared to the original Gltn-based adhesives [12–16]. Our results suggest that films composed of hm-Gltn have the ability to bond to soft tissues even under wet conditions. However, the detailed adhesive properties of these hm-Gltn films have not yet been clarified.

For this purpose, hydrophobically modified alkali-treated Gltns (hm-AlGltns) were prepared by the modification of the amino groups of AlGltn with fatty acid chlorides with various chain lengths, including hexanoyl (Hx: C_6_) chloride, decanoyl (Dec: C_10_) chloride, and stearyl (Ste: C_18_) chloride. We selected those fatty acid chlorides because our previous research showed that a longer hydrophobic group such as Ste resulted in good adhesivity to tunica media [13,15]. Therefore, the longest fatty acid is Ste in this research, and together we used a tunica blood vessel as an adherend, so, the fatty acid chlorides with different chain lengths could be compared with Ste. The obtained hm-AlGltns with various modification percentages were cast to fabricate films; these were then thermally crosslinked to prepare water-insoluble hm-AlGltn (t-hm-AlGltn) films. Using the t-hm-AlGltn films, surface wettability and bonding behavior on porcine blood vessels were evaluated.

## 2. Results and Discussion

### 2.1. Synthesis and Characterization of hm-AlGltns

**Figure 1.**
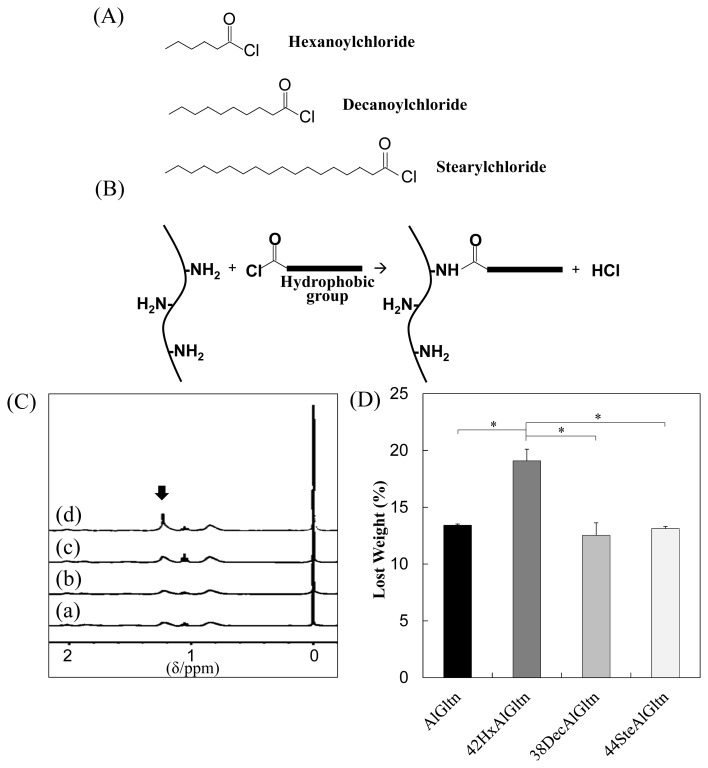
Modification of alkaline-treated gelatin (AlGltns) with hydrophobic groups using fatty acid chlorides and verification of the introduction. (**A**) Chemical formulas of fatty acid chlorides; and (**B**) nucleophilic substitution reaction between an amino group of the AlGltn molecule and a fatty acid chloride; (**C**) H^1^-NMR spectra of (a) AlGltn; (b) 42HxAlGltn; (c) 38DecAlGltn; and (d) 44SteAlGltn (the arrow indicates the peak derived for the methyl group of the alkyl chains); (**D**) Weight loss of hydrophobically modified alkaline-treated gelatin films (hm-AlGltns). Data are shown as the average ± S.D. of three samples (* *p* < 0.05).

As shown in Figure 1A, three different fatty acid chlorides, hexanoyl (Hx) chloride, decanoyl (Dec) chloride, and stearyl (Ste) chloride, were reacted with the amino groups of the AlGltn molecules by nucleophilic reactions to obtain HxAlGltn, DecAlGltn, and SteAlGltn.

Table 1 notes the characteristics of the hm-AlGltns after the nucleophilic substitution reactions of the amino groups of AlGltn with the fatty acid chlorides. The substituted quantities of amino groups with fatty acid chlorides were determined by the 2,4,6-trinitrobenzenesulfonic acid (TNBS) method [13–17]. Through this reaction, each hydrophobic group (Hx, Dec, Ste) was successfully introduced into each AlGltn with amide bonds to form hm-AlGltns, whose modification percentages ranged from 10% to 44%. Table 1 also lists the independent thermal denaturation temperature (*T*_d_) of each hm-AlGltn. The value of *T*_d_ of the 30% hm-AlGltns was lower than that of both the 10% and 40% hm-AlGltns. In addition, the hm-AlGltns with longer side chains exhibited a lower *T*_d_. The result may indicate that interaction between and coagulation of the introduced hydrophobic side chains together with the modest introduction ratio for crystallization, affected each *T*_d_.

Figure 1C shows the ^1^H-NMR spectra of the hydrophobic group of the molecule on the AlGltn or hm-AlGltns. The peak of the alkyl chain appears at 1.3 ppm. The peak intensities of the original AlGltn, 32HxAltn, 24DecAlGltn, and 26SteAlGltn at 1.3 ppm were 0.14, 1.48, 1.98, and 1.69. Therefore, each hydrophobic group was successfully introduced into each hm-AlGltn. On the other hand, the hm-AlGltns with longer side chains showed sharper ^1^H-NMR peaks.

The hydrated carbons of the longer side chains were easily detectable. Therefore, the 44SteAlGltn showed the sharpest peak as compared with other hm-AlGltns.

Weight losses of AlGltn, 42HxAlGltn, 38DecAlGltn, and 44SteAlGltn under heating from 25–300 °C were compared in Figure 1D. 42HxAlGltn showed the highest weight loss from 35 to 115 °C indicating that there is more bound water in 42HxAlGltn than AlGltn, 38DecAlGltn, and 44SteAlGltn. The higher weight loss means a looser network structure, each (hm-)AlGltn resulting from the AlGltn molecule of (hm-)AlGltn has higher flexibility.

### 2.2. Preparation of Thermally Crosslinked hm-AlGltn Films

Ten *w*/*v*% hm-AlGltn/HFIP solutions were cast and transparent hm-AlGltn films were fabricated. The films were subsequently heated at 140 °C *in vacuo* for thermal crosslinking. It is known that covalent amide crosslinks form between amino groups and carboxyl groups in Gltn molecules after thermal treatment of the gelatin membranes *in vacuo* [18–20], resulting in water-insoluble Gltn films. To evaluate the amino group amounts used for the thermal crosslinking, the 2,4,6-trinitrobenzenesulfonic acid (TNBS) method was employed and the results are given in Table 2. The hm-AlGltns with short side chains, such as 12HxAlGltn and 10DecAlGltn, consumed more amino groups than the original AlGltn during the thermal-crosslinking. The hm-AlGltns with dense and long side chains consumed fewer amino groups.

These results indicate that shorter side chains did not inhibit amide bond formation among the hm-AlGltns, whereas dense and longer side chains did because the amino group and carboxyl group on a Gltn molecule of an hm-AlGltn cannot exist owing to volume exclusion by inactive long side chains.

### 2.3. Mechanical Strength of Thermally Crosslinked hm-AlGltn Films

As shown in Table 2, the mechanical strength of the t-hm-AlGltns decreased with increasing modification percentage. Among t-hm-AlGltns with similar modification percentages but different alkyl chain lengths, the mechanical strength of films with longer side chains decreased. However, the t-12HxAlGltn and t-9DecAlGltn films were both stronger than the original t-AlGltn film.

It is hypothesized that with thermal crosslinking, the hm-AlGltn films acquired amide bonding between each hm-AlGltn molecule, thus becoming resistant to hydrolysis, even though dense hydrophobic groups inhibit the accessibility of the amino groups to the carboxyl groups of each hm-AlGltn molecule by volume exclusion. Amide bonds are more difficult to form in hm-AlGltns with dense hydrophobic groups than in hm-AlGltns with sparse hydrophobic groups.

These results are due to the fact that long hydrophobic groups inhibit the approach of amino groups to carboxyl groups, resulting in decreased crosslinking density in the t-hm-AlGltn films. As compared to the original AlGltn, hm-AlGltn molecules with low modification percentages can easily assemble by hydrophobic interaction. Therefore, the t-12HxAlGltn and t-9DecAlGltn films showed high stiffness as compared to the t-AlGltn film.

### 2.4. Water Content of Thermally Crosslinked hm-AlGltn Films

**Figure 2.**
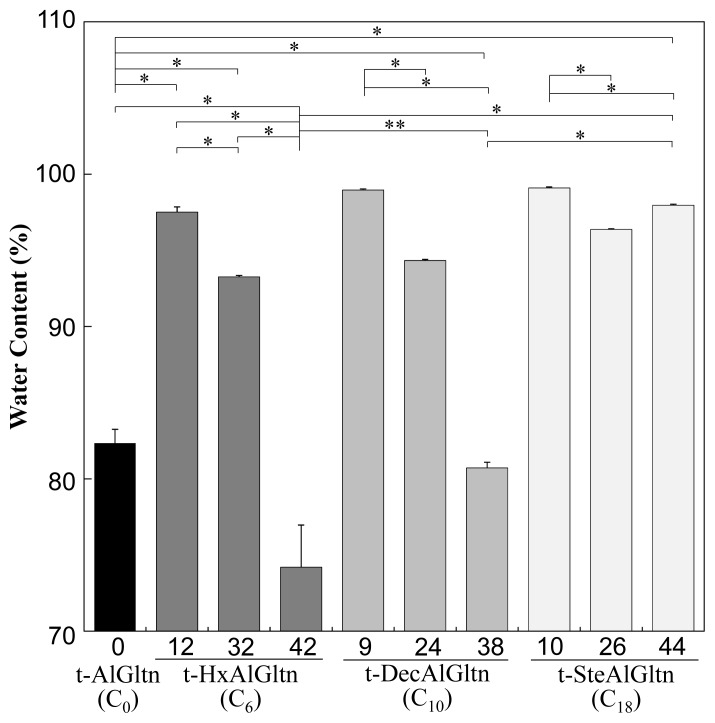
Water content of t-hm-AlGltn films after immersion in water at 37 °C for 5 min. Data are shown as the average ± S.D. of three samples (* *p* < 0.05, ** *p* ≥ 0.05).

Figure 2 shows the water content of each t-hm-AlGltn film after 5 min water immersion, at which point all the films reached a plateau. The water content of the films with dense side chains was lower compared to films with sparse side chains. On the other hand, the films with longer side chains showed higher water content compared to films with shorter side chains. The water content in t-9DecAlGltn and t-10SteAlGltn was high compared to the original t-AlGltn film. In all the prepared t-hm-AlGltn films, t-42HxAlGltn possessed the lowest water content.

These results indicate that long hydrophobic groups prevented the agglomeration of Gltn molecules due to volume exclusion, resulting in greater retention of water molecules. In contrast, the higher mobility of short hydrophobic groups promoted the agglomeration of Gltn molecules, leaving less room for water molecules in the film.

### 2.5. Surface Wettability of Thermally Crosslinked hm-AlGltn Films

When a film is applied for surgical use to close areas on organs, such areas will be in a wet condition because body fluids such as blood or lymph fluids ooze from the wound. Therefore, the films need to possess a high affinity for wet organ surfaces for use in surgical applications.

**Figure 3.**
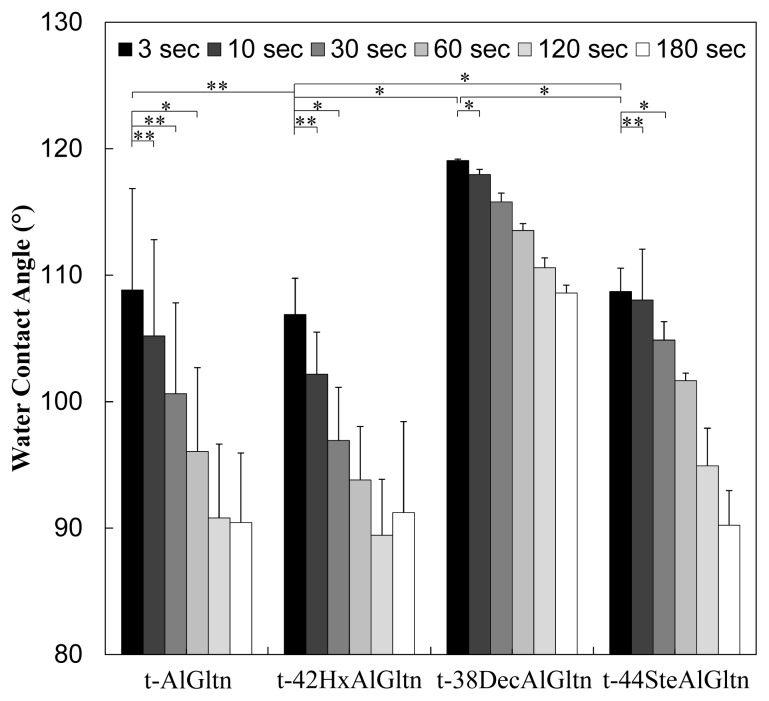
Surface wettability of t-hm-AlGltn films. Transition of static water contact angle on t-hm-AlGltn films (t-42HxAlGltn, t-38DecAlGltn, t-44SteAlGltn, and t-AlGltn). Data are shown as the average ± S.D. of three samples (* *p* < 0.05, ** *p* ≥ 0.05).

For the evaluation of wettability of t-hm-AlGltn films, the time dependence of the water contact angle was compared. In this experiment, films whose modification percentages were approximately 40%, *i.e.*, t-42HxAlGltn, t-38DecAlGltn, and t-44SteAlGltn, were used. As can be seen from Figure 3, the water contact angles of the samples decreased with time. Additionally each film shows independent wettability indicating that t-42HxAlGltn showed excellent wettability even though the amino groups of AlGltn were partially substituted by Hx. However, the wettability of t-38DecAlGltn and t-44SteAlGltn was quite low compared to t-AlGltn and t-42HxAlGltn.

Hx, the shortest chain possessing a low melting point of −3 °C, can move freely at 37 °C. Therefore, Hx can move more easily from the outside to the inside of the films to escape from the aqueous surface than can longer hydrophobic groups such as Dec and Ste, whose melting points are 31 °C and 69.6 °C, respectively.

### 2.6. Bonding Behavior of Thermally Crosslinked hm-AlGltn Films on the Porcine Blood Vessel

**Figure 4.**
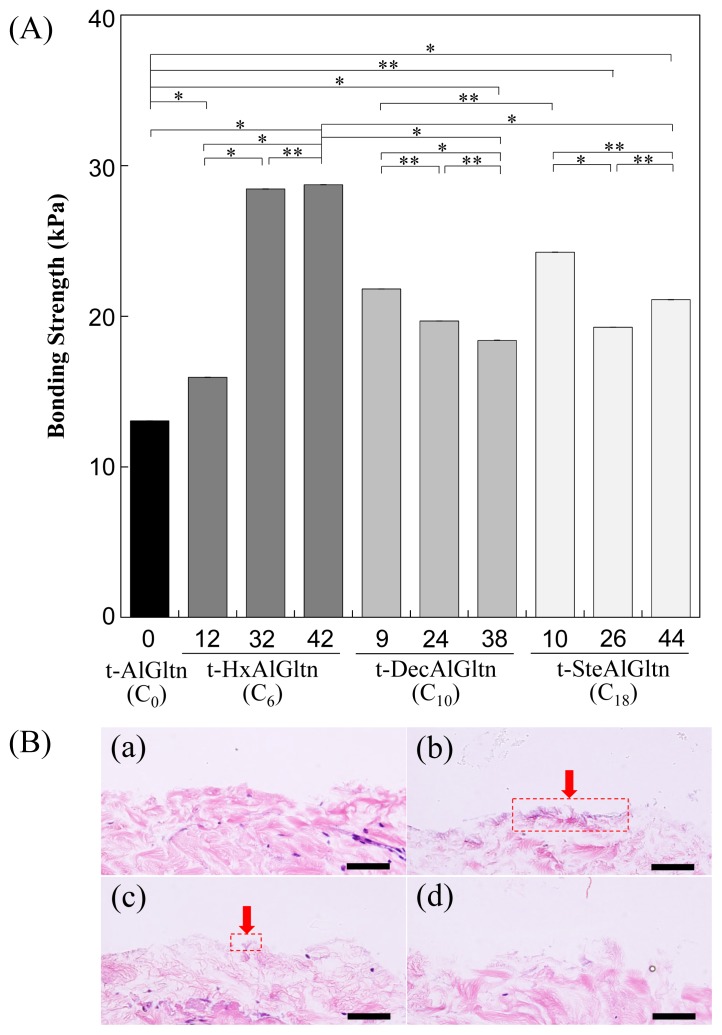
Bonding strength of t-hm-AlGltn films on the porcine blood vessel. (**A**) Effect of chain length and density of hm-AlGltns on the bonding strength. Data are shown as the average ± S.D. of three samples (* *p* < 0.05, ** *p* ≥ 0.05); (**B**) Cross-sectional views of the film-tissue interface after bonding strength measurement of (**a**) t-AlGltn; (**b**) t-42HxAlGltn; (**c**) t-38DecAlGltn; and (**d**) t-44SteAlGltn. Scale bar: 50 μm. Red arrows show the sites t-hm-AlGltn remained.

The bonding strength between the porcine blood vessel and t-hm-AlGltn films with various modification percentages is shown in Figure 4A. The bonding strength of all the t-hm-AlGltn films increased after the modification of the hydrophobic groups compared to the original t-AlGltn. In the case of the t-HxAlGltn films, the bonding strength increased with increasing modification percentage. The t-32HxAlGltn and t-42HxAlGltn films in particular had bond strengths that were 2.5 times greater than that of the t-AlGltn film. The modification percentages of t-DecAlGltn and t-SteAlGltn did not show sufficient enhancement of bond strength even though their bonding strengths were greater than that of the t-AlGltn film. This result indicates that the Hx group is the most effective side chain for blood vessel adhesion among the three hydrophobic groups, Hx, Dec, and Ste. The Hx group could easily interpenetrate the hydrophobic region of the extracellular matrix (ECM) or the hydrophobic amino acid and cell membrane of the tissue because of its low melting point, resulting in its higher mobility. This interpenetration of Hx contributed to the high bonding strength of t-42HxAlGltn to the porcine blood vessel. Furthermore, Gltn molecules in t-42HxAlGln have the ability to partially form a triple helix with the Gltn molecule collagen on the surface of the blood vessel.

After measurement of the bonding strength, all of the tissue-film interfaces were fixed and hematoxylin and eosin (HE) staining was applied to observe the bonding/destruction behavior of the t-hm-AlGltn films on porcine blood vessels. By HE staining, the film remaining on the tissue surface and the tissue itself were colored dark purple and pink, respectively. Figure 4B(a,d) show that no t-AlGltn or t-44SteAlGltn film remained on the blood vessel surfaces. However, the t-42HxAlGltn and t-38DecAlGltn films (Figure 4B(c,d), indicated by red arrows and dashed rectangles) clearly remained after the bonding strength measurement. Additionally, the remaining t-42HxAlGltn was thicker and broader than the t-38DecAlGltn, indicating the higher penetration ability of the Hx group into the blood vessel as compared with the Dec group.

Recently, we reported that a tissue adhesive composed of cholesteryl group-modified Gltn and disuccinimidyl tartrate showed excellent bonding ability onto porcine arterial media [14–16]. From histological observations, a thick adhesive layer was found on the media surface even after the bonding strength measurement. In the present case, only a thin adhesive layer was observed. The t-hm-AlGltn films seemed to be much stiffer than the cholesteryl group-modified Gltn-based adhesive; therefore, film remaining on the blood vessel surface could be hard even when the films were bonded strongly.

### 2.7. Cell Adhesion onto t-hm-AlGltn Films

To compare cell adhesivity onto the films, L929 cells were seeded onto t-AlGltn, t-42HxAlGltn, t-38DecAlGltn, and t-44SteAlGltn films. After being cultured for 5 min, the number of adhered cells was counted. Also, the morphology of the adhered cells was observed using a scanning electron microscope (SEM).

**Figure 5.**
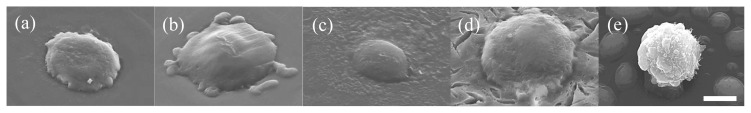
L929 adhesion onto t-hm-AlGltn films after being cultured for 5 min. Morphology of L929 cells on t-hm-AlGltn films ((**a**) t-AlGltn; (**b**) t-42HxAlGltn; (**c**) t-38DecAlGltn; (**d**) t-44SteAlGltn and (**e**) tissue culture plate). Scale bar: 5 μm.

Figure 5 shows SEM images of L929 cells adhered onto t-hm-AlGltn films or culture plate dishes after being cultured for 5 min. The cells on the tissue culture plate show a spherical shape (Figure 5e). However, the cells on each t-(hm-)AlGltn surface, especially on t-42HxAlGltn surface, spread more extensively on the t-42HxAlGltn surface (Figure 5a–d), indicating that short side chains such as Hx could easily interact with the cell membrane.

Many more L929 cells adhered on t-42HxAlGltn than on t-AlGltn, t-38DecAlGltn, and t-44SteAlGltn (data is not shown). The obvious difference may come from the easy Hx interpenetration into the cell membrane due to its low melting point.

**Figure 6.**
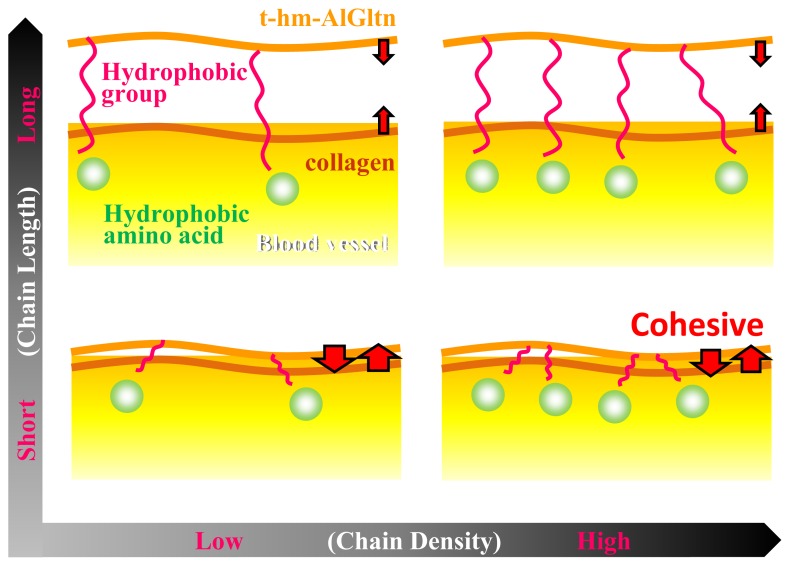
Schematic illustration of the bonding behavior of t-hm-AlGltns with various hydrophobic chain lengths and densities.

The results indicate that the introduced hydrophobic group Hx interacts with the tissue components, including collagen and the cell membrane. This means that the Hx group penetrated into the hydrophobic domains of the tissue, such as the hydrophobic amino acid residue and the phospholipids of the cell membrane. Figure 6 shows the effect of the chain length and the density on the ability of t-hm-AlGltn molecules to bond onto a blood vessel. When long chains such as those of the Ste and Dec groups are introduced into the AlGltn molecules, the resulting hm-AlGltn cannot easily interact with collagen molecules on the surface of the blood vessel because of their higher melting points and volume exclusion. Also, longer chains with lower mobility can hardly interact with hydrophobic groups because of hydrophobic amino acids in blood vessels. On the other hand, t-42HxAlGltn molecules with dense and short chains with a lower melting point easily interact with blood vessels and form stronger bonds.

In this study, t-24DecAlGltn and t-38DecAlGltn were employed for comparison. If DecAlGltns with a slightly higher introduction ratio like t-30DecAlGltn and t-40DecAlGltn were compared, the bonding strength would be weaker than t-24DecAlGltn and t-38DecAlGltn. The hypothesis is from our previous data that hm-Gltns with a much higher introduction ratio, over 50% introduction ratios, bonded more weakly than t-hm-Gltns with appropriate introduction ratios. The higher amount of alkyl chains can agglomerate by hydrophobic interaction in the film, therefore, there will be less hydrophobic group which can interact with the hydrophobic group in the ECM of the blood vessel.

## 3. Experimental Section

### 3.1. Materials

BeMatrix™, an alkaline-treated gelatin (AlGltn) derived from porcine skin, was kindly donated by Nitta Gelatin Inc. (Osaka, Japan). Ethanol (EtOH), 1,1,1,3,3,3-hexafluoroisopropanol (HFIP), dimethylsulfoxide (DMSO), triethylamine (TEA), 2,4,6-trinitrobenzoylsulfonic acid (TNBS), hydrochloric acid (HCl), sodium dodecyl sulfide (SDS), calcium chloride, 10% formalin neutral buffer solution, 4′,6-diamidino-2-phenylindole (DAPI), tris(hydroxymethyl)aminomethane, tert-butylalcohol, and glycine were purchased from Wako Pure Chemical Industries, Ltd. (Osaka, Japan). Hexanoyl (Hx: C_6_) chloride, decanoyl (Dec: C_10_) chloride, and stearyl (Ste: C_18_) chloride were purchased from Sigma Chemical Co. (St. Louis, MO, USA). A porcine aorta was purchased from Funakoshi Corporation (Tokyo, Japan). L929 cells were purchased from (RIKEN Bio Resource Center Cell Bank, RBRC-RCB2619, Ibaraki, Japan). All chemicals were used without further purification.

### 3.2. Synthesis of hm-AlGltns

Based on former reports [13–16], hm-AlGltns with various chain lengths and densities were prepared by the reaction between fatty acid chlorides and primary amino groups of AlGltn. The employed fatty acid chlorides were hexanoyl (Hx: C_6_), decanoyl (Dec: C_10_), and stearyl (Ste: C_18_) chloride. First, AlGltn (10 g) was fully dissolved into 99 mL of dried DMSO at 80 °C. Then, one mL of TEA was added into the AlGltn/DMSO solution to obtain 100 mL of 10 *w*/*v*% AlGltn/DMSO solution under a dry N_2_ atmosphere. The fatty acid chloride was subsequently added to the AlGltn solution and stirred for 17 h at room temperature. The resulting hm-AlGltn/DMSO solution was then poured into 300 mL of cold EtOH and stirred for 1 h. Subsequently, the precipitate of hm-AlGltn was washed twice with 300 mL of cold EtOH followed by evaporation under vacuum to leave a white cake of which the yield was calculated.

### 3.3. Characterization of hm-AlGltns

The modification percentages of the hydrophobic groups in AlGltn were quantified by the method previously reported using TNBS [13–17]. Briefly, each hm-AlGltn and the original AlGltn were dissolved in DMSO to obtain 0.05 *w*/*v* % solutions. Then, 100 μL of 0.1 *v*/*v* % TEA/DMSO, 50 μL of 0.1 *w*/*v* % SDS/DMSO, and 100 μL of 0.1 *w*/*v* % TNBS/DMSO were added to 100 μL of each (hm-) AlGltn/DMSO solution, followed by incubation at 37 °C for 2 h under light-shielding conditions. Then, 50 μL of the 2 *N*-HCl/DMSO solution was added to stop the reaction. Finally, the intensity of light absorbance was measured spectrophotometrically at 340 nm using a microplate reader (GENios A-5082, Tecan Japan, Kanagawa, Japan). The substitution percentage of amino groups with the fatty acid chlorides was then calculated from the intensities of hm-AlGltn compared with the original AlGltn.

The modification of the fatty acid in AlGltn was confirmed by ^1^H-NMR (AL300, JEOL, Tokyo, Japan) and FT-IR (FTIR-8400S, Shimadzu, Kyoto, Japan) measurements. The typical peaks were found at 2357 cm^−1^ (C=O bond of long-chain fatty acids) and 2332–2323 cm^−1^ (C–N bond of amino bonding between fatty acids and the amino groups of the AlGltn molecules).

Thermogravimetry (TG) analysis was executed to analyze thermal behavior of obtained hm-AlGltns (TG8120, Rigaku, Tokyo, Japan). Heating was conducted from 30 to 300 °C at a heating rate of 10 °C/min. Aluminium oxide was employed as control.

The thermal behavior of the hm-AlGltn solution was also analyzed by differential thermal analysis (DSC) (DSC8230, Rigaku, Tokyo, Japan). The hm-AlGltns were dissolved in ultrapure water (Merck Millipore, Tokyo, Japan) to prepare 70 *w*/*v*% samples. Heating was conducted from 0 to 100 °C at a heating rate of 5 °C/min under nitrogen atmosphere.

### 3.4. Preparation and Characterization of Thermally Crosslinked hm-AlGltn Films

Each hm-AlGltn was first dissolved in HFIP to prepare a 10 *w*/*v* % solution. Each solution (2.5 mL) was then cast on a 4 × 4 × 0.5 cm mold with a glass plate bottom and a silicone wall and was dried for 12 h, followed by drying overnight under vacuum at room temperature. The resulting films were placed between two thin silicone sheets and sandwiched between metal plates. Thermal treatment was then performed under vacuum at 140 °C for 24 h.

In order to determine the residual amino group amount in t-hm-AlGltn, each film was cut into a disk 4 mm in diameter and then immersed in 300 μL of ultrapure water. Then, 300 μL of a 4 *w*/*v* % NaHCO_3_ aqueous solution and 300 μL of a 0.1 *w*/*v* % TNBS aqueous solution were added. After incubation for 2 h, 600 μL of 6 *N*-HCl was added to stop the reaction. Then, 300 μL of each sample was placed into each well of a 96-well plate and the absorbance was measured at 340 nm using a microplate reader (GENios A-5082, Tecan Japan, Kanagawa, Japan).

### 3.5. Measurement of Water Content of Thermally Crosslinked hm-AlGltn Films

The thermally crosslinked hm-AlGltn films were cut into disks 4 mm in diameter and were immersed in 1 μL of ultrapure water at 37 °C. The weights of the swollen films after various time periods were gravimetrically determined. The water content of the films was calculated using the following equation:

(1) $$\text{Water content }(\%)=\frac{W^{\prime }-W}{W^{\prime }}\times 100$$

*W*′: weight of swollen film; *W*: weight of dried film.

The water contents of the films after 5 min immersion in water were compared.

### 3.6. Determination of Surface Wettability of Thermally Crosslinked hm-AlGltn Films

In order to determine the surface wettability of the thermally crosslinked hm-AlGltn films, the water contact angle of each film was measured using a contact angle meter (DM800, Kyowa Interface Science Co., Ltd., Saitama, Japan). Briefly, 2 μL of ultrapure water was placed on the t-hm-AlGltn films and the time dependent change in the static water contact angle was measured after 3, 10, 30, 60, 120 and 180 s. The resulting data were analyzed using FAMAS software (Kyowa Interface Science Co., Ltd., Saitama, Japan).

### 3.7. Measurement of the Mechanical Strength of Thermally Crosslinked hm-AlGltn Films

Each film was cut out into 5 × 1 mm rectangular shapes and both sides were bonded on 5 mm × 1 cm plastic sheets by GelBoy (LOCTITE, Henkel Japan, Tokyo, Japan) in a 2.5 × 5 mm area. After drying at room temperature, the plastic sites were clipped to probes and tensile tests were performed for all samples before and after thermal crosslinking at a rate of 10 mm/min (*n* = 3).

### 3.8. Measurement of Bonding Strength

There was no existing protocol in place to evaluate the bonding strength between the tissue surface and the film; therefore, the following measurement method was applied. The porcine blood vessel was dissected with a dermal punch into disks 4 mm in diameter. The dissected blood vessel was bonded onto a probe with GelBoy. The t-hm-AlGltn films were also punched out into 7 mm diameter disks and placed on a heated plate at 37 °C. They were fixed to the heated plate with scotch tape (3M, Tokyo, Japan) with a hole 4 mm in diameter. The bonding strength was then measured using a Texture Analyzer (TA-XT2i, Stable Micro Systems, Godalming, UK) (*n* = 3) with the following conditions: 180 s contact time, 20 g/mm^2^ applied force, and 10 mm/min tracking speed.

### 3.9. Observation of t-hm-AlGltn Film–Blood Vessel Interfaces

After the bonding strength measurement, each sample was fixed with a 10% formalin neutral buffer solution followed by hematoxylin and eosin (HE) staining. Cross sections of the stained samples were observed with an optical microscope (BX51, Olympus, Tokyo, Japan).

### 3.10. Cell Adhesion onto t-hm-AlGltn Film

A mouse fibroblast cell line, L929, was used to evaluate cell adhesion onto the t-hm-AlGltn films. The L929s were first cultured in a medium (RPMI-1640 (R8758, Sigma-Aldrich, St. Louis, MO, USA)) containing 2 *v*/*v* % fetal bovine serum. The t-hm-AlGltn films were placed on 24-well plates and a glass ring was put on each film. L929 cells (5.0 × 10^4^ cells) were seeded onto each film for 5 min and the films were rinsed with 2 mL of phosphate buffered saline (PBS, pH 7.4). Then the cells were fixed with 10% formalin neutral buffer solution for 60 min and permeabilized in 0.2 *v*/*v* % Triton-X 100 in PBS for 2 min followed by 0.1% DAPI in PBS for 10 min in light-shielding conditions at room temperature. The adhered cells were observed with an IX81 inverted fluorescence microscope (Olympus Co. Ltd., Tokyo, Japan). The counted number of adhered cells was calculated from the area of the microscopic field (*n* = 3).

The cells were then observed with a scanning electron microscope (SEM). In brief, the cells were gradually dehydrated with a 50–99 *v*/*v* % ethanol/water solution. Then, the cells were immersed in tert-butylalcohol twice followed by freeze drying at −80 °C. The cells were then observed by SEM.

### 3.11. Statistical Analysis

Statistical analysis was carried out using Student’s *t*-test with Microsoft Excel software. Statistically significant differences were accepted when *p* <0.05. The data are shown as mean ± standard deviation (S.D.).

## 4. Conclusions

Thermally crosslinked film adhesives composed of hydrophobically modified AlGltn with Hx (C_6_), Dec (C_10_), or Ste (C_18_) were fabricated and their bonding behaviors on porcine blood vessels were evaluated. The t-42HxAlGltn film with short and dense hydrophobic groups showed higher wettability, lower water content, and stronger bonding to the blood vessel compared to the other t-hm-AlGltn films.

The t-42HxAlGltn and t-38DecAlGltn films remaining after bonding strength measurement were confirmed by histological observation. L929 cells rapidly adhered and extended onto the t-42HxAlGltn film compared with other films. These results indicate that the t-42HxAlGltn film has potential for biomedical applications as a film adhesive.

## Figures and Tables

**Table 1.** Hydrophobically modified alkaline-treated gelatin films (hm-AlGltns) with various modification ratios.

Abbreviation	Number of carbons	Fatty acid chloride		Modification (%)	Yield (%)	*T*_d_ (ºC)

		in feed (μL)	in amino groups of AlGltn (%)			
12HxAlGltn	6	16	10	12	91	36.2
32HxAlGltn	6	49	30	32	93	34.2
42HxAlGltn	6	82	50	42	95	36.5
10DecAlGltn	10	409	50	10	76	36.8
24DecAlGltn	10	818	100	24	78	35.4
38DecAlGltn	10	409	50	38	89	36.9
10SteAlGltn	18	110	10	10	85	37.1
26SteAlGltn	18	330	30	26	55	36.5
44SteAlGltn	18	550	50	44	91	39.6

The prefixes of the abbreviations indicate the reacted ratio of amino group of AlGltn molecule with fatty acid chloride and the introduced hydrophobic group (Hx, Dec, or Ste): 12HxAlGltn means that 12% of amino group of AlGltn molecule was reacted with Hx chloride.

**Table 2.** T-hm-AlGltns with various modification percentages.

Abbreviation	Number of carbons	Amino groups used for thermal crosslinking (%)	Stiffness in dried state (MPa)
t-AlGltn	0	14.9 ± 6.3	4.66 ± 0.22
t-12HxAlGltn	6	30.7 ± 5.9	5.39 ± 0.05
t-32HxAlGltn	6	11.0 ± 4.9	4.39 ± 0.20
t-42HxAlGltn	6	10.1 ± 1.9	3.68 ± 0.60
t-10DecAlGltn	10	20.0 ± 2.6	5.06 ± 0.11
t-24DecAlGltn	10	3.5 ± 0.5	4.24 ± 0.29
t-38DecAlGltn	10	0.2 ± 2.0	3.06 ± 0.06
t-10SteAlGltn	18	2.9 ± 0.9	4.01 ± 0.11
t-26SteAlGltn	18	7.2 ± 0.1	3.49 ± 0.42
t-44SteAlGltn	18	3.5 ± 1.4	2.19 ± 0.24
